# Minimally Invasive Coronary Artery Bypass Grafting in a Patient With Chronic Tracheostoma: Alternative to Reduce Sternal Wound Complication Risk

**DOI:** 10.1177/15569845221137898

**Published:** 2022-12-26

**Authors:** Alex Nantsios, Elsayed Elmistekawy, Menaka Ponnambalam, A. Stephane Lambert, Marc Ruel

**Affiliations:** 1 Division of Cardiac Surgery, University of Ottawa Heart Institute, ON, Canada

**Keywords:** minimally invasive, coronary artery bypass grafting, tracheostomy

## Abstract

Patients with chronic tracheostoma present a challenge when they require coronary bypass surgery due to an elevated risk of sternal wound infections (SWI). Minimally invasive coronary artery bypass grafting (MICS CABG) is a robust technique that allows complete surgical revascularization while mitigating the risks of sternal complications and functional decline associated with sternotomy. In such patients at elevated risk for SWI, MICS CABG may represent a viable revascularization strategy to avoid sternotomy. Here, we present a case of a frail, comorbid patient with a chronic tracheostomy and symptomatic multivessel coronary artery disease not amenable to percutaneous therapy referred for MICS CABG.

## Introduction

Patients with chronic tracheostomas present a challenge when they require cardiac surgery due to concerns for sternal wound infection (SWI). Tracheostomy, when employed postoperatively, is an independent predictor of SWI, with a risk reported up to 18.6%,^1^ thought to be related to the close proximity of the nonsterile stoma to the surgical wound. Similarly, a preexisting tracheostoma theoretically imposes a considerable risk for infection in patients undergoing sternotomy for myocardial revascularization.^2^ Previously, authors have attempted to maintain the distance from the tracheostomy site, using alternative approaches including the “T-modified” manubrium-sparing median sternotomy^3^ or the “Figure L approach,” which consists of an anterior thoracotomy extended vertically downward to the peritoneal cavity.^4^

Minimally invasive coronary artery bypass grafting (MICS CABG) via a small anterior thoracotomy is a robust technique that allows complete surgical revascularization, providing excellent outcomes,^5^ while circumventing the risk of sternal wound complications. In such patients at elevated risk for SWI, MICS CABG may represent a viable revascularization strategy to avoid sternotomy. Here, we present a case of a patient with a chronic tracheostoma with symptomatic multivessel coronary artery disease not amenable to percutaneous therapy, undergoing MICS CABG.

## Case Report

**Fig. 1.**
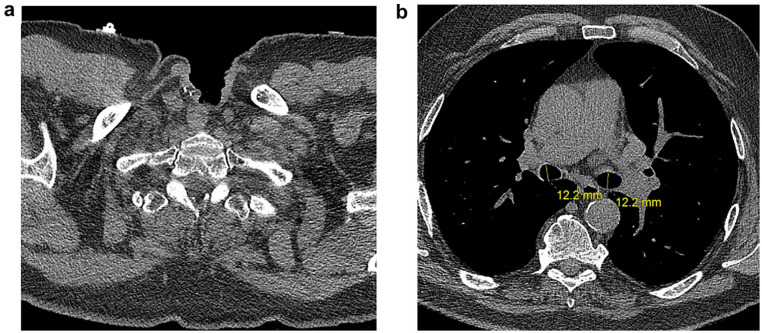
Preoperative non-contrast CT chest study depicting patent tracheal stoma and tracheobronchial tree. (a) CT slice demonstrating prior laryngectomy and tracheostoma. (b) Mainstem bronchi with normal caliber, left measuring 12 × 15 mm and right measuring 12 × 18 mm. CT, computed tomography.

A 76-year-old male patient with chronic tracheostoma after prior oropharyngeal and laryngeal resections and previous percutaneous coronary intervention (PCI) to the left anterior descending (LAD) artery, presented with Canadian Cardiovascular Society Class IV angina due to severe distal left main stenosis (70%) at a trifurcation, as well as 90% mid-LAD and 70% left circumflex artery stenoses, with nonobstructive disease in the right coronary artery (Supplemental Video 1). All lesions were hemodynamically significant by instant wave-free ratio. He had previously undergone neck radiotherapy in 2006 for tonsillar squamous cell carcinoma and later developed primary laryngeal carcinoma, treated with pharyngolaryngectomy, thyroidectomy, and soft-tissue reconstruction in 2015. Due to osteoarthritis, the patient also had significant functional impairment and used a cane to mobilize. He was frail with a Clinical Frailty Scale score of 5. He was not a candidate for PCI due to high anatomic risk. He was deemed ineligible for sternotomy by the surgical team at a referring outside institution. He was referred for second opinion and was consented for MICS CABG. A computed tomography scan was obtained (Fig. 1) to assess the caliber of the tracheobronchial tree, to allow for left lung isolation.

Under light sedation and topical anesthesia of the trachea, a fiber-optic bronchoscopy confirmed patency of the airway. The stoma was cannulated with a #9.0 armored endotracheal tube. Following induction of general anesthesia, the endotracheal tube was upsized to size #9.0, and an Arndt endobronchial blocker (Cook Medical, Bloomington, IN, USA) was inserted through the endotracheal tube into the left mainstem bronchus and carefully adjusted using fiber-optic bronchoscopy. The patient was positioned in a 15° to 30° right lateral decubitus position with the left arm on a padded arm rest. The surgical field was prepped and draped, exposing the left anterolateral thorax, sternum, and bilateral groins. The bronchial blocker was inflated, and adequate lung isolation was confirmed. A 4 cm left thoracotomy was performed in the fourth interspace at the midclavicular line. The left internal thoracic artery (LITA) was harvested in a nonskeletonized fashion, and the saphenous vein (SVG) was harvested from the right leg. Heparin was administered, and the pericardium was incised anterolaterally. A proximal anastomosis was performed to the ascending aorta after application of a side-biting clamp. Distal anastomosis was performed positioned with Medtronic Octopus® Nuvo Tissue Stabilizer and Starfish® Evo Heart Positioner (Medtronic, Dublin, Ireland). To optimize exposure of the lateral wall, the shaft was removed from the Starfish positioner, and an umbilical tape tied around its base was used to apply traction toward the patient’s right hip.^6^ The SVG was anastomosed to the obtuse marginal artery (Supplemental Video 2), with a flow of 35 mL/min and pulsatility index of 1.5 confirmed with a transit-time flow meter (Medistim, Oslo, Norway). The LITA was then anastomosed to the LAD with flow of 18 mL/min and pulsatility index of 2. Protamine was administered, hemostasis was achieved, and the wound was closed in multiple layers.

**Fig. 2.**
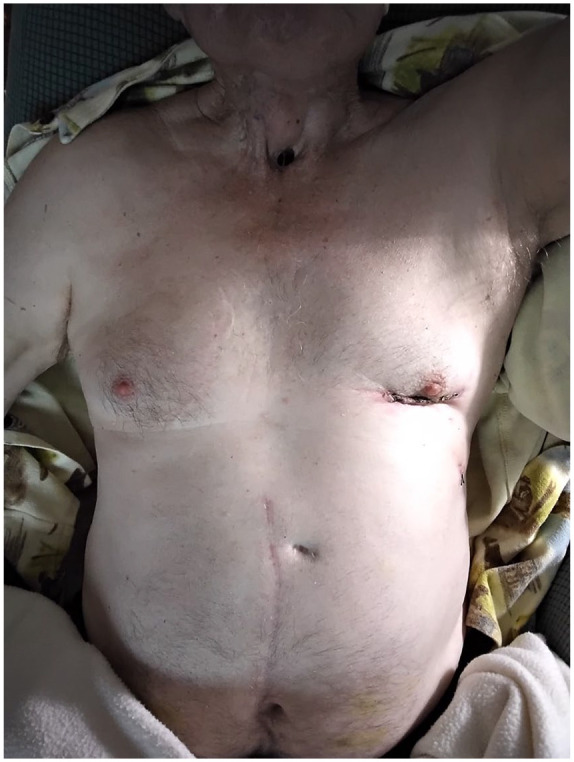
Healed small left thoracotomy wound in relation to the chronic tracheostoma.

Postoperatively, the patient was extubated within 6 h after the operation and transferred to the floor on the first postoperative day. He required 30% to 40% FiO_2_ through humidified air into the tracheal stoma until postoperative day 3. Intense physiotherapy and secretion management was instituted. The patient was discharged from the hospital on the fourth postoperative day with independent mobility, given the lack of physical restrictions. There was no evidence of wound infection upon clinical follow-up at 3 weeks (Fig. 2). Informed consent to report patient information and images was obtained.

## Discussion

MICS CABG is an effective technique achieve complete revascularization, while decreasing the invasiveness of conventional CABG. MICS CABG offers many benefits including shorter hospital length of stay, faster recovery to full activity,^7^ and minimization of wound complications, with a reported SWI rate of 0.2%.^5^ For these reasons, it is preferred in frail patients with significant comorbidity, who possess strong indications for surgical myocardial revascularization.

Patients with chronic tracheostomies impose a large theoretical risk of SWI and mediastinitis, which is associated with higher operative mortality, leading to hesitancy to recommend sternotomy. In addition, sternotomy increases risks of stoma necrosis and tracheal injuries. Given the distance of the thoracotomy wound from the colonized tracheostoma, MICS CABG essentially nullifies the risk of infection in these patients.

In the case presented here, a comorbid patient with tracheostoma (2 cm above the sternal notch) presented with a complex left main lesion not amenable to PCI. Sternotomy was deemed high risk due to an elevated risk of SWI and mediastinitis. Importantly, this patient also had several markers of frailty, which is a known contributor to cardiac surgical risk that is not often incorporated in traditional risk calculators. Furthermore, frailty is a strong predictor of prolonged hospitalization and disability.^8^ With advancement in minimally invasive techniques, there should be increasing focus on the preoperative assessment of frailty to target optimal management of these patients. Using the technique of MICS CABG, this patient with elevated risk of SWI and several markers of frailty received complete revascularization including LITA-LAD with a short hospital stay and without prolonged functional compromise.

A tracheostomy should not preclude multivessel surgical myocardial revascularization. MICS CABG is a safe and effective approach to minimize the risk of infection and functional disability after sternotomy. Although experience is limited, this alternative approach warrants further evaluation in this group of patients.

## Supplemental Material

Visual abstract – Supplemental material for Minimally Invasive Coronary Artery Bypass Grafting in a Patient With Chronic Tracheostoma: Alternative to Reduce Sternal Wound Complication Risk Click here for additional data file. Supplemental material, sj-pptx-1-inv-10.1177_15569845221137898 for Minimally Invasive Coronary Artery Bypass Grafting in a Patient With Chronic Tracheostoma: Alternative to Reduce Sternal Wound Complication Risk by Alex Nantsios, Elsayed Elmistekawy, Menaka Ponnambalam, A. Stephane Lambert and Marc Ruel in Innovations: Technology and Techniques in Cardiothoracic and Vascular Surgery
