# Supplementary material for: Minimally Invasive Coronary Artery Bypass Grafting in a Patient With Chronic Tracheostoma: Alternative to Reduce Sternal Wound Complication Risk
Source: Innovations (Phila). 2022 Dec 26;17(6):574–6. doi: 10.1177/15569845221137898 (PMC9846372; doi:10.1177/15569845221137898)
Supplement: Visual abstract – Supplemental material for Minimally Invasive Coronary Artery Bypass Grafting in a Patient With Chronic Tracheostoma: Alternative to Reduce Sternal Wound Complication Risk [file sj-pptx-1-inv-10.1177_15569845221137898.pptx]

## Slide 1
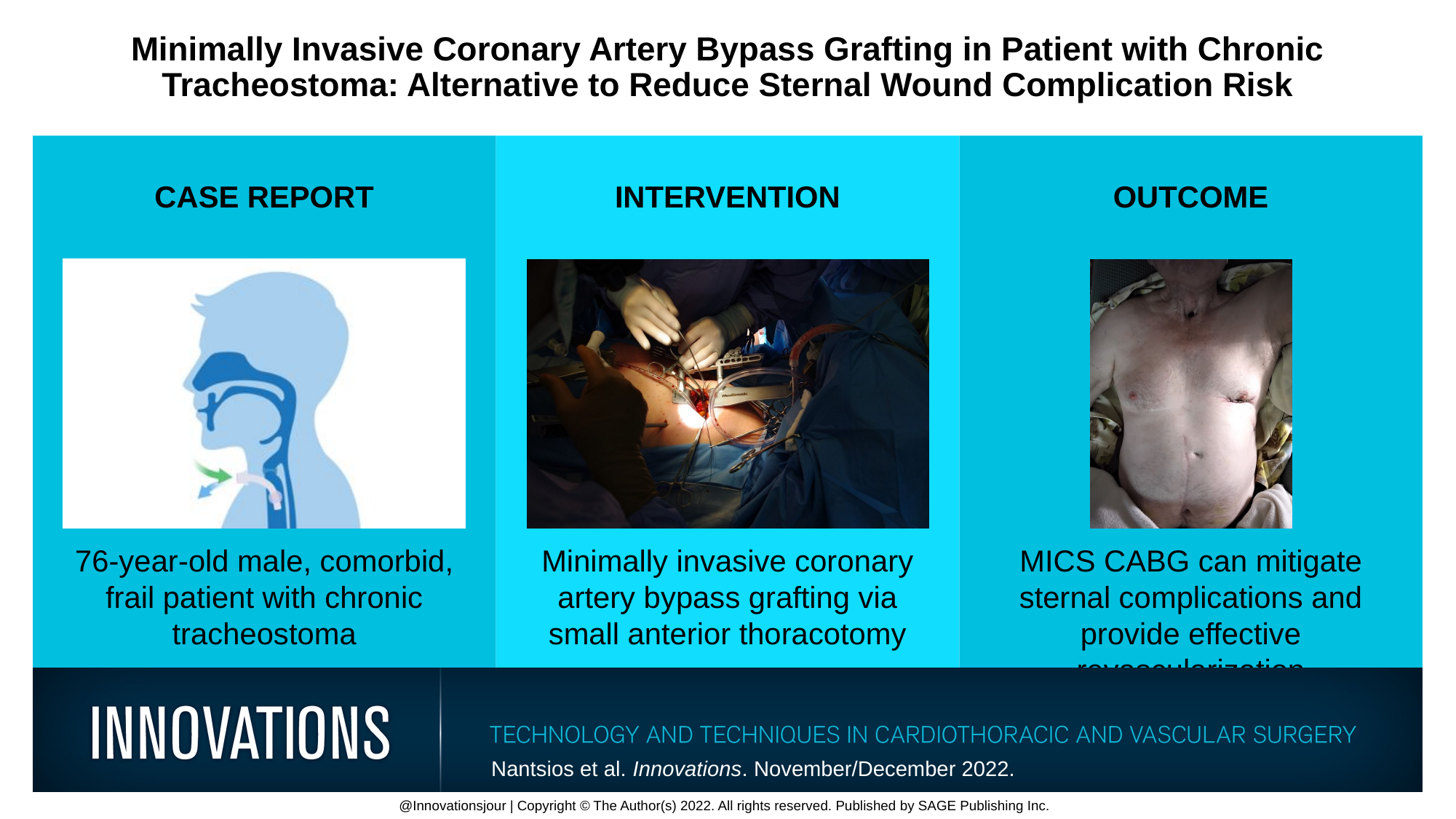

# Minimally Invasive Coronary Artery Bypass Grafting in Patient with Chronic Tracheostoma: Alternative to Reduce Sternal Wound Complication Risk
CASE REPORT
76-year-old male, comorbid,
frail patient with chronic tracheostoma
INTERVENTION
Minimally invasive coronary artery bypass grafting via
small anterior thoracotomy
OUTCOME
MICS CABG can mitigate sternal complications and provide effective revascularization
Nantsios et al. Innovations. November/December 2022.
@Innovationsjour | Copyright © The Author(s) 2022. All rights reserved. Published by SAGE Publishing Inc.
